# Sex and the Developmental Environment Shape Molecular Networks Underlying Bronchial Responsiveness in Mice

**DOI:** 10.1096/fj.202503280R

**Published:** 2026-02-10

**Authors:** Razia Zakarya, Baoming Wang, Yik Lung Chan, Dikaia Xenaki, Kin Fai Ho, Hai Guo, Hui Chen, Brian G. Oliver, Christopher O'Neill

**Affiliations:** ^1^ School of Life Sciences University of Technology Sydney Sydney Australia; ^2^ Epigenetics of Chronic Disease Group, Woolcock Institute of Medical Research, Macquarie University Sydney Australia; ^3^ Respiratory Cell and Molecular Biology Group, Woolcock Institute of Medical Research Macquarie University Sydney Australia; ^4^ Jockey Club School of Public Health and Primary The Chinese University of Hong Kong, Hong Kong Special Administrative Region of the People's Republic of China Hong Kong People's Republic of China; ^5^ Air Quality Studies, Department of Civil and Environmental Engineering The Hong Kong Polytechnic University Hong Kong People's Republic of China

## Abstract

Epidemiological evidence supports sex‐specific prevalence patterns of respiratory disease, yet the molecular basis of these dimorphic patterns under normal physiological conditions remains poorly understood. Using an isogenic murine model, we assessed bronchial responsiveness to methacholine in male and female adult offspring, with and without maternal exposure to air pollution particulates. We confirmed that males exhibit significantly greater bronchial responsiveness than females, independent of maternal exposure. RNA sequencing of lung tissue, coupled with gene co‐expression analysis, revealed differentially expressed genes and sex‐specific gene network modules associated with this physiological dimorphism. Interestingly, although maternal exposure did not alter the physiological response, it did interact with sex to affect which gene modules are associated with bronchial responsiveness. These findings provide new insight into the molecular architecture of sex‐based differences in lung function and highlight the importance of incorporating both sex and developmental context in respiratory research.

## Graphical abstract

Sex and the developmental environment shape molecular networks underlying bronchial responsiveness in mice. A methacholine challenge was performed on 13‐week‐old isogenic male and female offspring (+/− developmental particulate matter exposure) to show that males had significantly higher bronchial responsiveness (red arrows) than females. Lung transcriptomic analysis showed differential expression and co‐expression modules underlying bronchial responsiveness between sexes, with maternal exposure interacting with sex to affect gene networks underlying bronchial response to methacholine.

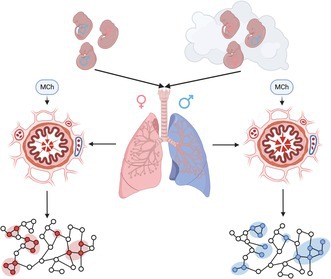

**Abbreviations**
BR: Bronchial Responsiveness
COPD: Chronic Obstructive Pulmonary Disease
CPM: Counts Per Million
DEG: Differentially Expressed Gene
eQTL: Expression Quantitative Trait Loci
GO: Gene Ontology
GSEA: Gene Set Enrichment Analysis
GWAS: Genome Wide Association Study
NIH: National Institute of Health
PM_2.5_
: Particulate Matter ≤ 2.5 μm in diameter
SABV: Sex as a Biological Variable
TRANSFAC: Transcription Factor database
WGCNA: Weighted Gene Co‐expression Network Analysis

## 1 Introduction

Biological sex influences lung development, immune function, and susceptibility to respiratory disease [1, 2], yet the molecular basis of these differences—particularly under physiological conditions—remains poorly defined. While hormonal and chromosomal factors can play an important role, developmental exposure to environmental toxins may also modulate sex‐specific transcriptional programs [3]. Here, we position this study within the broader context of sex‐linked respiratory phenotypes and the need to better understand how early‐life and biological factors intersect to shape lung function.

In eutherian mammals, biological sex is determined by the paternal sex chromosome—X or Y—delivered by the male gamete. Fusion with the maternal X chromosome produces XX (female) or XY (male) offspring. Gonadal sex is driven by the presence or absence of the *Sry* gene on the Y chromosome [4, 5, 6]. *Sry* initiates signaling pathways [7] that direct testis development and Sertoli cell differentiation [4, 6, 8], while its absence allows for the gonads to develop into ovaries [5, 9, 10]. In both cases, sex‐specific gene networks guide gonadal differentiation, triggering hormonal cascades that shape external genitalia [11]. Resultant male (XY) and female (XX) chromosomal complements drive distinct transcriptional programs underpinning sex‐specific gonadal and phenotypic differentiation.

Although developmental differences between the male and female sexes are vast, it was only in 2014 when the National Institute of Health (NIH) announced its intention to urge funding applicants to consider sex as a biological variable (SABV) [12]. The implementation of the SABV policy was underpinned by numerous robust associations showing sex as an independent variable affecting disease prevalence [13, 14, 15], response to therapeutics [16, 17], and clinical outcomes [15, 18]. With respect to pathologies of the lung, sex‐specific prevalence patterns are present in asthma [2], chronic obstructive pulmonary disease (COPD) [1, 19, 20], and lung cancer [21]. Interestingly, asthma prevalence exhibits age‐dependent sexual dimorphism, with higher rates in males before puberty and in females thereafter [22, 23, 24]. Such patterns are affected by an intersection of genes, hormones, and environmental exposures unique to each sex, but this report will focus solely on sex‐specific gene expression.

While biological sex is a key determinant of transcriptional programming, it is also well established that the maternal environment can shape gene expression patterns in offspring. For example, we have shown that maternal exposure to air pollution insult can induce increased transcriptional entropy in asthmatic adult offspring [25]. However, it remains unclear whether and how these environmentally induced transcriptional changes interact with sex‐linked gene regulatory networks. Addressing this gap is critical to understanding the origins of sex‐specific disease vulnerability, particularly in organs like the lung that exhibit dimorphic physiology and disease burden.

Studies have shown that there are transcriptomic patterns unique to biological sex in numerous pathological conditions. Examples specific to the lung include fibrotic pulmonary myeloid cells [26], malignancies [27, 28], and response to harmful environmental pollutants such as smoking [29]. To adequately unpick whether this gene expression difference is attributed to etiology or sequelae of the respective pathology, it is important to determine which sex‐based transcriptomic differences occur during healthy conditions. Numerous multi‐tissue studies have shown sex‐based transcriptomic differences unique to tissue types in humans [30, 31, 32, 33] and mice [34] but few have linked sex‐specific gene expression profiles to biologically occurring dimorphic phenotypes. One example of such a study [35] integrated expression quantitative trait loci (eQTL) information from genome‐wide association studies (GWAS) to show that a cluster of sex‐specific gene expression was associated with height‐increasing effects in men and a separate cluster with height‐decreasing effects in females [35]. Other studies have shown sex‐stratified eQTLs initiated upon glucocorticoid receptor activation were associated with risk for psychiatric disorders [36] and sex‐specific transcriptome changes associated with dimorphic differences in cardiomyocyte contractility [37].

In the lung, it has been shown that non‐malignant human tissue [38], human foetal tissue at the pseudoglandular stage of development [3], neonatal hyperoxic lung injury [39], non‐small cell lung cancer [27], and human airway epithelium from smokers [29] show bulk transcriptomic sexual dimorphism. However, there are no studies to date that integrate sex‐based transcriptomic changes with a biologically occurring dimorphic phenotype occurring under normal physiological conditions in the lung.

A well characterized example of a dimorphic physiological response in the lung would be the bronchial responsiveness (BR) to an inhaled constrictor agonist, such as methacholine [40, 41, 42]. Here, we investigate this sex‐based difference in BR under normal physiological conditions, with and without maternal exposure to air pollution. By integrating physiological assessment with bulk transcriptomic profiling, we identify sex‐specific gene expression patterns and co‐expression modules associated with this dimorphic trait.

## 2 Materials & Methods

### 2.1 Air Pollution Particulate Collection

Air pollution particulate matter ≤ 2.5 μm in diameter (PM_2.5_) was collected as previously described [43]. Briefly, in the summer, PM_2.5_ was collected from a busy roadside in Hong Kong, extracted via sonication and freeze‐dried overnight. Organic carbon, elemental carbon, and water‐soluble inorganic ions were determined, as previously published [25].

### 2.2 Animal Experiments

Animal experiments were approved by the Animal Care and Ethics Committee of the University of Technology Sydney (ETH18‐3175). All protocols were performed according to the Australian National Health and Medical Research Council Guide for the Care and Use of Laboratory Animals. Female BALB/c mice ( *Mus musculus* ) (6 weeks, Animal Resources Centre, Perth, WA, Australia) were housed at 20°C ± 2°C and maintained on a 12 h light, 12 h dark cycle (lights on at 06:00 h) with ad libitum access to standard rodent chow and water; all animals were acclimatized for 1 week prior to the commencement of experimentation. As previously described [25], female BALB/c mice were divided into two groups, Sham (*n* = 15) and PM_2.5_ (*n* = 15) dams. The PM_2.5_ dams were exposed to PM_2.5_ standardized to Sydney, Australia levels [44, 45] (5 μg/day intranasal exposure) suspended in 40 μL saline prior to mating for 6 weeks, during the entirety of gestation (~3 weeks), and during weaning (~3 weeks). The Sham dams were exposed to saline (0.9% NaCl; 40 μL in total, 20 μL each naris) for the same period. Our PM_2.5_ dam treatment schedule recapitulates maternal exposure to air pollution prior to mating, during gestation and through lactation. Offspring were never directly exposed to PM_2.5_, however, they risk exposure to those components of PM_2.5_ that pollute lactation, as has been shown in real world studies [46]. As such, our model of real‐world maternal exposure to the offspring is termed the “developmental window” of exposure.

### 2.3 Bronchial Responsiveness Measurement

FlexiVent (SCIREQ, Montreal, QC, Canada) apparatus was used to measure bronchial responsiveness in response to increasing doses of methacholine (Sigma‐Alrich, St Louis, MO, USA) using the forced oscillation technique. Briefly, male and female offspring were anesthetized (tribromoethanol, 250 mg/kg, intraperitoneally, Sigma‐Aldrich, St Louis, MO, USA) to prevent spontaneous breathing and tracheostomised. Then, an 18‐gauge polyethylene cannula was inserted. The cannula was connected to the flexiVent and ventilated at 200 breaths/min with a tidal volume of 10 mL/kg and a positive end‐expiratory pressure of 3 cm H_2_O.

Lung function was performed at baseline and after increasing doses of methacholine (0, 1.6, 3.125, 6.25, 12.5, and 25 mg/mL, Sigma‐Alrich, St Louis, MO, USA) generated by the Aeroneb Lab vibrating‐mesh nebulizer (Aerogen Ltd., Galway, Ireland) integrated within the flexiVent system (SCIREQ Inc., Montreal, Canada). The small‐particle head (2.5–4.0 μm VMD) was used, operating with a 50% duty cycle (2 s on/2 s off) for approximately 10 s per methacholine dose to measure airway reactivity. Deep Inspiration maneuvers were performed to a maximum pressure of 30 cm H_2_O and held for 3 s, as per the manufacturer's default settings. Two Deep Inspirations were performed before each dose to standardize the volume history. The impedance of the respiratory system was analyzed using the constant phase model to calculate Newtonian resistance (Rn, reflecting airway resistance). Following each methacholine nebulization (small particle head 2.5–4.0 μM VMD, Aeroneb Lab Nebulizer; Aerogen Ltd., Galway, Ireland), QuickPrime‐3 perturbations (3 s each) were triggered every 10 s to monitor dynamic airway responses. The maximal airway response (Rn) was defined as the average of three consecutive measurements showing the highest values. Lung samples were collected after completion of the lung function testing.

### 2.4 Tissue Homogenization & RNA Extraction

A 10 mg portion of snap frozen right lower lobe lung from 13‐week‐old male and female offspring was homogenized using the PrecellysTM Tissue Homogenizer (Bertin Technologies, IDF, France). Sample mRNA was extracted using the AllPrep DNA/RNA/Protein Mini Kit (QIAGEN, Hilden, Germany) as per the manufacturer's instructions. Quantification of mRNA was carried out with a NanoDrop2000 (Themofisher, North Ryde, Australia).

### 2.5 Library Preparation and Sequencing

Extracted RNA samples (Male = 11 Sham & 14 PM; Female = 11 Sham & 13 PM) from offspring underwent quality control (QC), library prep and RNA sequencing at the Ramaciotti Centre at UNSW Sydney. RNA libraries were sequenced as 100 bp single‐end reads on Illumina NovaSeq 6000.

### 2.6 Data Handling

Reads were trimmed with Trimmomatic (version 0.39), mapped to GRCm38 with Bowtie2 (version 2.5.1) and SAMtools (version 1.17), and log2‐transformed reads per million were quantified and statistically analyzed (DESeq) using SeqMonk (v1.48.1; Babraham Institute). See statistics methods for details on statistical analysis.

### 2.7 Statistics

#### 2.7.1 Bronchial Responsiveness

Statistical analysis for lung function tests was performed using IBM SPSS Statistics Package (version 27.0.1.0). All data were tested for normality and appropriate statistical models were applied accordingly. BR data were analyzed using Univariate Analysis of Variance with Sex, (Maternal) Treatment group, and Methacholine concentration as fixed factors and Tukey's post hoc test. All data are presented as mean ± standard error of mean unless otherwise stated. All researchers were aware of the group allocation at all stages of the project.

#### 2.7.2 Differential Gene Expression

Data evaluation and statistical analysis were performed using Seqmonk (v1.48.1; Babraham Institute). To define differentially expressed genes (DEGs), DESeq2 was applied to raw counts, with QC metrics and treatment included as covariates and a threshold of *p* < 0.01 after correction for multiple comparisons with the Benjamini‐Hochberg analysis.

#### 2.7.3 Weighted Gene co‐Expression Network Analysis (WGCNA)

We performed WGCNA in R (v4.3.1) using the WGCNA package (v1.72‐1) to identify modules of co‐expressed genes associated with bronchial responsiveness (Rn) at 25 mg/mL methacholine and area under the curve (RnAUC), sex, and maternal exposure.

#### 2.7.4 Input Data and Pre‐Processing

Gene expression data from individual offspring lungs and corresponding phenotype intersected by sample ID. Traits included Rn, sex, and maternal exposure. Samples and genes were quality filtered.

#### 2.7.5 Network Construction and Module Detection

Soft‐thresholding power (R^2^ > 0.8) was selected to approximate scale‐free topology. A signed co‐expression network was constructed, and modules were assigned unique color labels. A gene dendrogram with module colors was plotted.

#### 2.7.6 Module–Trait Association and Visualization

Module eigengenes were correlated with traits using Pearson correlation, with *p* values computed and FDR‐adjusted using the Benjamini–Hochberg method. For each significant module, genes were extracted and eigengene–trait scatterplots were generated, stratified by sex or maternal exposure where appropriate.

#### 2.7.7 Gene Set Enrichment Analysis and Visualization

Enrichment analysis was performed on DEGs using g:Profiler (version e113_eg59_p19_f6a03c19, database updated on 23/05/2025) across Gene Ontology (GO: molecular function), TRANSFAC, and miRtarBase biological pathways with enrichment threshold set at *p* < 0.05. Outputs were visualized with R (v 4.3.1) package ggplot2 (v 3.5.1).

## 3 Results

### 3.1 Biological Sex Affects BR in Adult Mice

**FIGURE 1:**
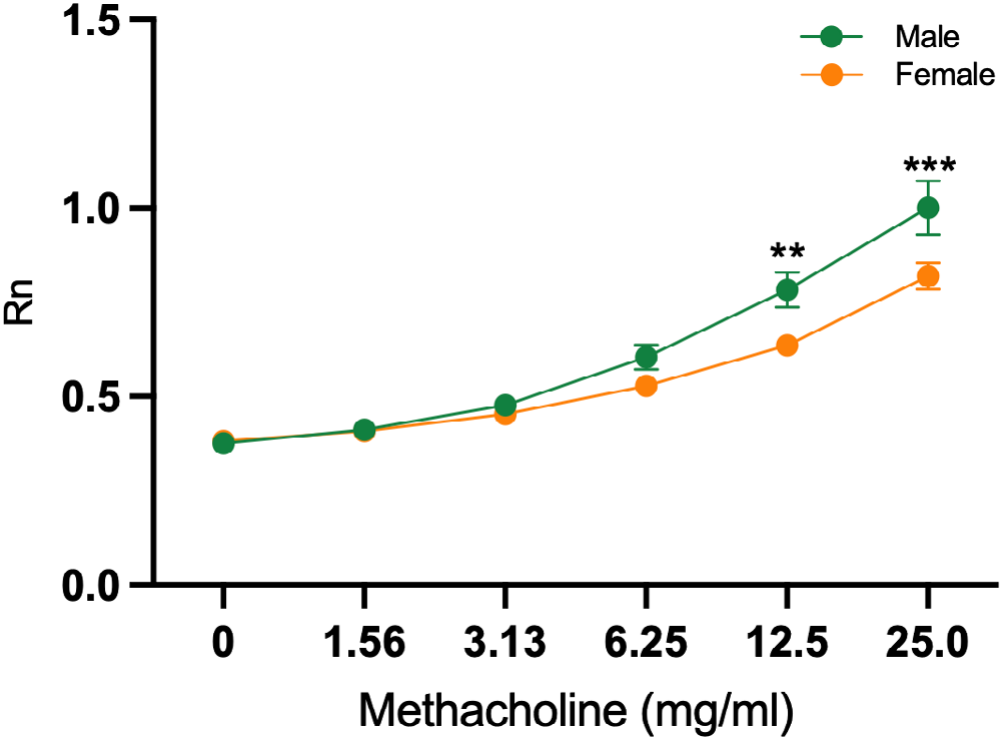
Spasmogen challenge and lung function testing using FlexiVent lung function measurement performed on 13‐week‐old offspring shows that increasing doses of methacholine increase airway resistance (Rn), with males (*n* = 25) showing significantly higher resistance than females (*n* = 24; **p < 0.001, ***p < 0.0001).

We exposed 13‐week‐old female (Sham *n* = 11; PM *n* = 13) and male (Sham *n* = 11; PM *n* = 14) offspring mice to increasing doses of aerosolised methacholine to determine the effect of the spasmogen on airway resistance. Methacholine induced a dose‐dependent increase in airway resistance, with no significant interaction effect between sex and maternal exposure (Figure S1). The Sham and PM groups were therefore pooled for each sex to increase statistical power (females *n* = 24; males *n* = 25). Sex and methacholine concentration did show a significant interaction effect (Table S1). Male mice exhibited significantly greater dose‐dependent BR than females (Figure 1), with this effect becoming evident from 12.5 mg/mL methacholine. To investigate molecular mechanisms underlying this sex‐specific response, we performed bulk RNA‐seq on lung tissue from the same cohort (females *n* = 24; males *n* = 25).

### 3.2 Biological Sex Affects Pulmonary Gene Expression

To account for any sex‐specific differences in cellular composition, we performed cellular deconvolution (supplementary methods) of male and female gene expression counts per million (CPM). This analysis showed that there was no significant difference in the cellular distribution profile between male and female lung samples (Figure S2). Differential gene expression analysis using DESeq at a statistical stringency of *p* < 0.01 and |log2 fold change| > 0.6 found male lungs to have 67 differentially expressed genes (DEGs) when compared to isogenic female lungs of the same age (Table S2). Maternal treatment was included as a covariate and showed no significant effect. Of these, 23 were downregulated in males, whilst 44 were upregulated in males. It is worth noting that only 8 of these genes were located on the sex chromosomes (Table 1).

**TABLE 1:** List of sex chromosome specific DEGs.

Gene	Chromosome	Log2FC
*Gm29650*	Y	8.05610371
*Uty*	Y	10.035532
*Ddx3y*	Y	10.9341183
*Kdm5d*	Y	10.9573507
*Eif2s3y*	Y	11.4654331
*Xist*	X	−5.9059267
*Xlr4a*	X	−1.310257792
*Kdm6a*	X	−0.667500317

### 3.3 Sexually Dimorphic DEGs Are Enriched Across Numerous Gene Ontology Functions

**FIGURE 2:**
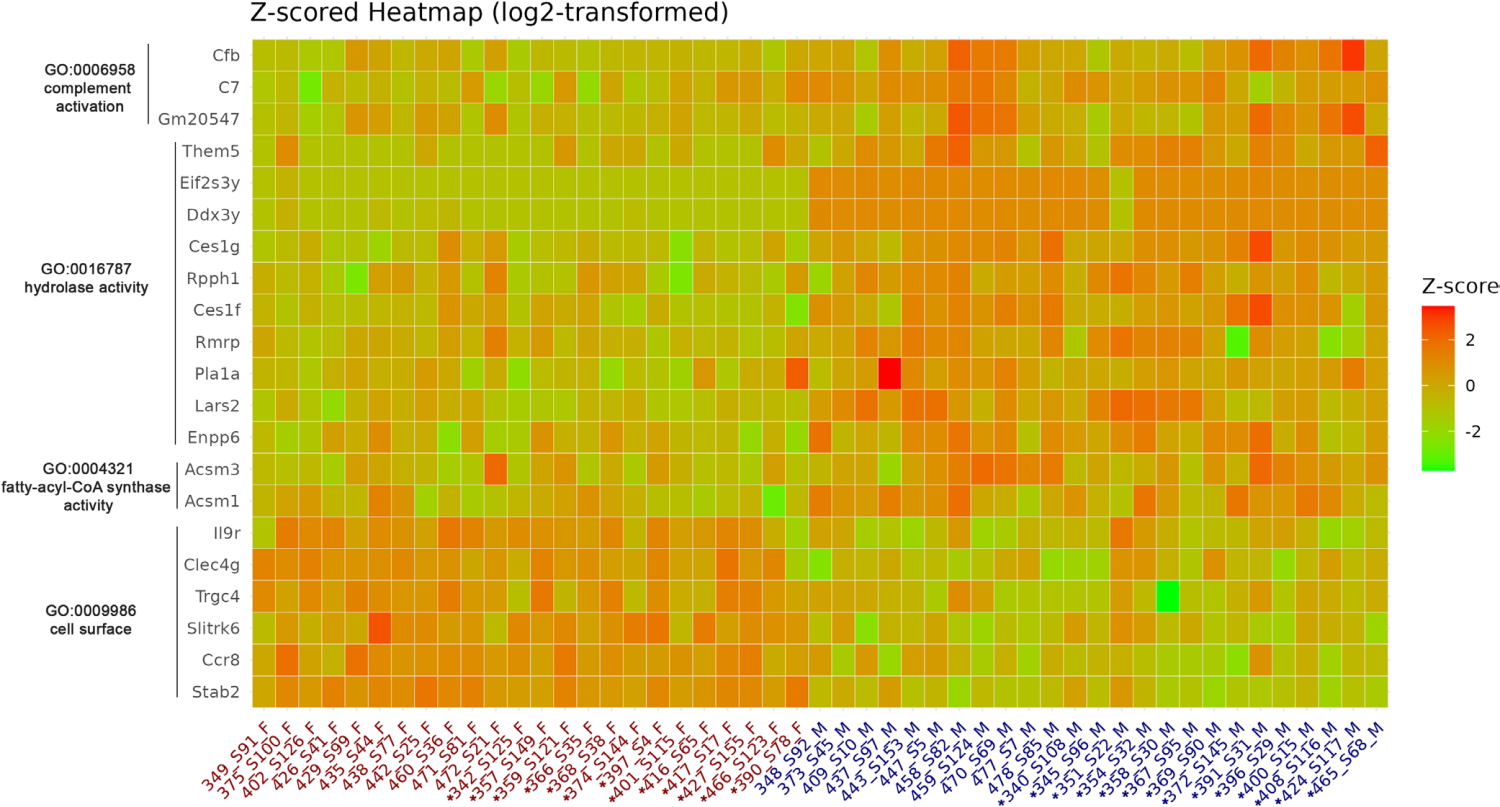
Differentially expressed genes when comparing male (navy) and female (red) lung show enrichment across numerous GO pathways (*denotes maternal PM_2.5_ treatment). Gene expression data are presented as a z‐score of log2‐transformed Counts Per Million.

We performed Gene Set Enrichment Analysis (GSEA) of DEGs across Gene Ontology (GO), KEGG, and Reactome data libraries. Those genes downregulated in males did not enrich along KEGG or Reactome biological pathways but were enriched across GO functions associated with cell surface and plasma membrane pathways, with notable effector genes including the downregulation of *Stab2, Ccr8, Slitrk6, Trgc4, Clec4g*, and *Il9r* (Figure 2) in males. Genes upregulated in males were enriched in pathways associated with hydrolase activity, fatty‐acyl‐CoA synthetase activity, and complement activation, with key gene drivers shown in Figure 2.

We also performed GSEA across regulatory motifs compiled within the TRANScription FACtor database (TRANSFAC) and miRTarBase, which respectively inform upon enrichment across transcription factor binding sites & pathways and microRNA‐Target interactions. This analysis showed no enrichment of sex‐based DEGs across common regulatory motifs curated across TRANSFAC and miRTarBase.

### 3.4 Sex‐Influenced Gene Co‐Expression Networks Reveal Distinct Molecular Drivers of Bronchial Reactivity

In addition to identifying sex‐specific DEGs, we sought to determine whether distinct molecular pathways underlie BR in males and females. To investigate this, we applied Weighted Gene Co‐expression Network Analysis (WGCNA) to construct a unified gene co‐expression network and identify modules—clusters of genes with highly correlated expression profiles (Figure S3).

Module eigengenes were correlated with airway resistance (Rn) values at the 25 mg/mL methacholine dose, incorporating sex and maternal treatment as biological variables. Both factors were included because each had a significant independent effect on Rn (Table S1); although the maternal treatment × sex interaction was non‐significant, maternal treatment remained an important main contributor to variance in Rn. We further assessed whether sex or maternal exposure modulated the relationship between gene modules and BR by testing for interaction effects (e.g., Rn × Sex, Sex × Maternal Exposure). This enabled the identification of gene networks exhibiting sexually dimorphic expression, as well as those whose associations with BR were sex‐ or maternal exposure‐dependent.

**FIGURE 3:**
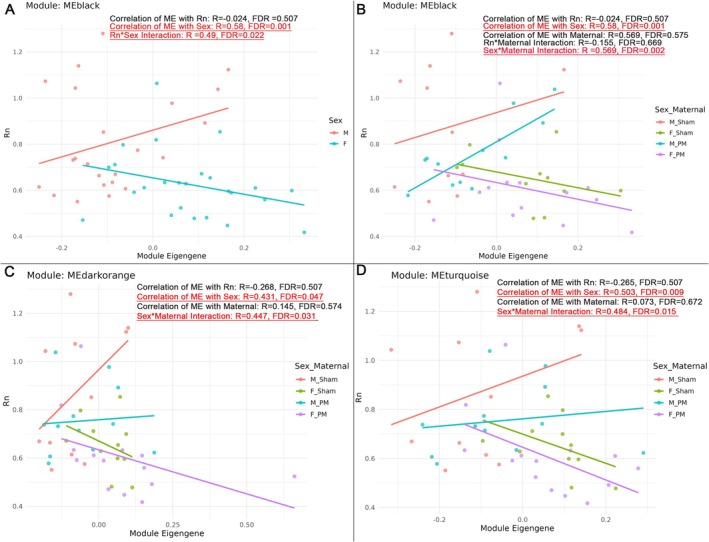
Sex and Sex × Maternal interactions modulate module‐trait associations. (A) MEblack shows a significant association with sex (FDR = 0.001) and a sex‐dependent relationship with Rn (Rn × Sex, FDR = 0.022) and (B) a sex × maternal interaction (FDR = 0.002). (C) MEdarkorange is associated with sex (FDR = 0.047) and sex × maternal interaction (FDR = 0.031). (D) MEturquoise is associated with sex (FDR = 0.009) and sex × maternal interaction (FDR = 0.015). Each panel shows module eigengene (x‐axis) versus airway resistance (Rn, y‐axis) for *n* = 47 offspring. Lines denote linear fits by sex or sex × maternal group. Red font denotes significant interaction (FDR > 0.05).

After correcting for multiple testing using the Benjamini–Hochberg method, no gene co‐expression modules were significantly associated with airway resistance (Rn) or maternal exposure alone. However, three modules—MEblack (354 genes), MEdarkorange (86 genes), and MEturquoise (4917 genes)—exhibited sexually dimorphic expression patterns and were significantly influenced by interactions between sex and maternal treatment (Figure 3a–d). Notably, MEblack also showed a significant sex‐dependent association with Rn, suggesting that the relationship between airway responsiveness and this module's gene expression is moderated by biological sex.

We assessed whether individual genes within each of the three significant modules showed particularly strong correlations with the external traits (i.e., high kME) that might indicate potential hub genes or leading drivers. However, no single gene within MEblack, MEdarkorange, or MEturquoise exhibited a notably stronger association than the others, suggesting that the module–trait relationships are distributed across the network rather than driven by a single dominant gene.

Module eigengenes correlated with area under the curve resulted in a loss of any significant sex effect, whilst retaining RnAUC × Sex and Sex × Maternal Treatment in module ME Black and Sex × Maternal Treatment in ME Green. Substituting RnAUC for Rn reduced the statistical significance of sex effects, indicating that the interaction patterns we observed are driven primarily by responses at the most discriminatory methacholine dose, rather than the integrated airway response.

### 3.5 Module Gene Sets Enriched Along Numerous Key Pathways

**FIGURE 4:**
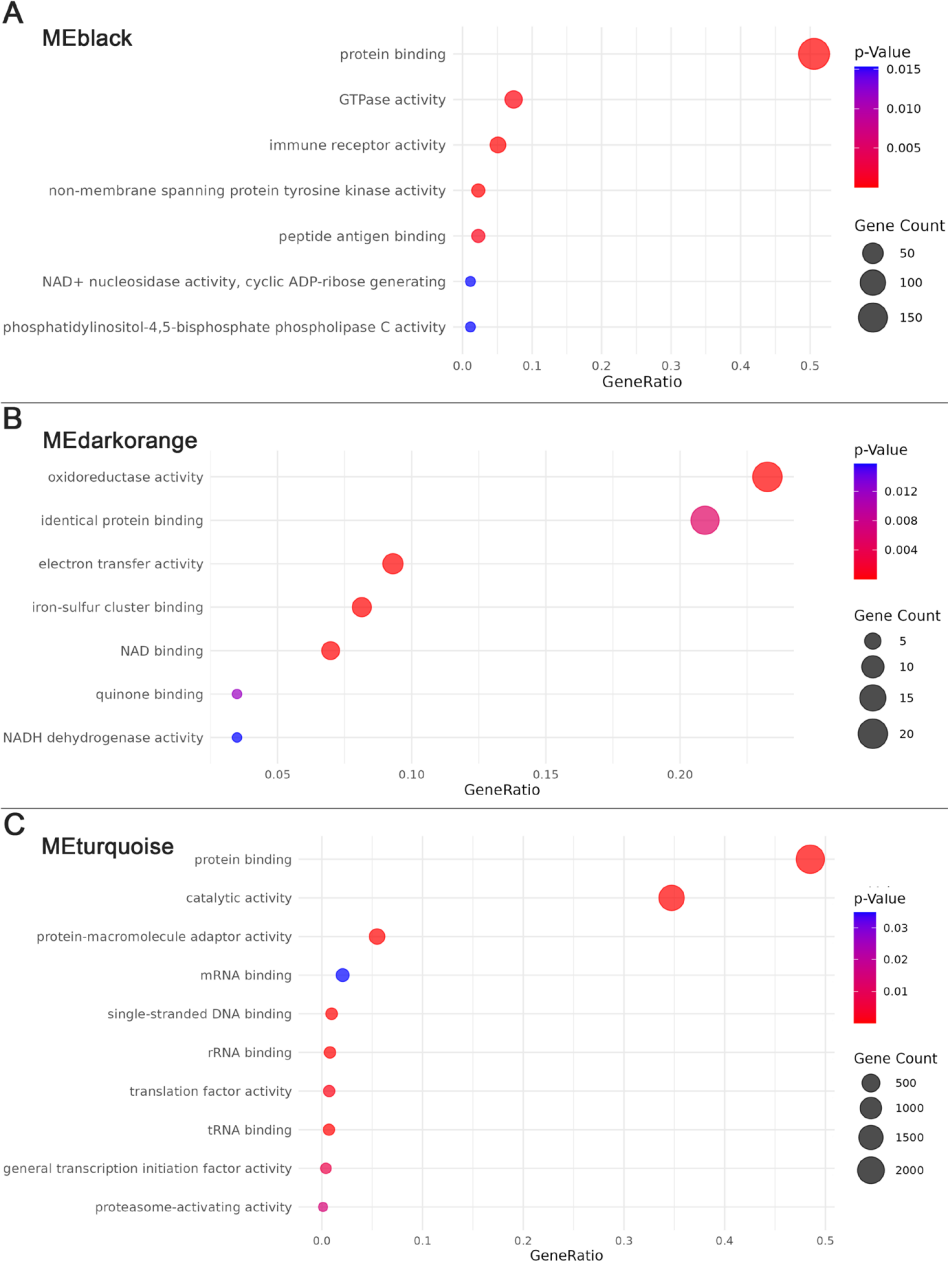
Functional enrichment of sex‐ and interaction‐associated gene co‐expression modules for (a) MEblack, (b) MEdarkorange, and (c) MEturquoise. Gene Ontology (GO) terms (Molecular Function) were identified using over‐representation analysis. Dot size indicates the number of genes in each enriched term, and color represents statistical significance (Benjamini–Hochberg adjusted *p* values).

To gain functional insight into the biological roles of the sex‐ and interaction‐associated modules, we performed GSEA against the GO molecular function aspect for genes within MEblack, MEdarkorange, and MEturquoise. Each module exhibited enrichment in biologically relevant pathways (Figure 4).

MEblack (Figure 4a) was enriched for immune‐related and signaling functions, including *immune receptor activity, peptide antigen binding*, and *non‐membrane spanning protein tyrosine kinase activity*, as well as broader functions such as *protein binding* and *GTPase activity*. These enrichments are consistent with immune modulation as a potential sex‐specific regulator of airway reactivity.

MEdarkorange (Figure 4b) showed strong enrichment for mitochondrial redox and electron transfer pathways, including *oxidoreductase activity, electron transfer activity, iron–sulfur cluster binding*, and *NADH dehydrogenase activity*. These findings suggest that mitochondrial and metabolic gene networks may contribute to sex‐differential airway physiology.

MEturquoise (Figure 4c) was significantly enriched in RNA‐binding and translational control pathways, including *mRNA binding, rRNA binding, tRNA binding*, and *translation factor activity*, indicating a potential post‐transcriptional regulatory program responsive to an interaction between sex and the developmental environment.

Collectively, these enrichments highlight the distinct biological programs captured by each module and support the notion that sexually dimorphic and interaction‐sensitive networks influence airway function through diverse molecular mechanisms.

### 3.6 Integration of Differential Expression and Co‐Expression Modules

To integrate insights from both differential expression and gene network analyses, we cross‐referenced sex‐associated DEGs with genes assigned to modules identified via WGCNA. We specifically examined whether any of the DEGs were present within modules whose eigengenes were significantly associated with sex or sex‐based interactions.

Although MEdarkorange was significantly associated with sex, it did not contain any overlapping DEGs. In contrast, MEblack contained four sex‐associated DEGs (*Trgc4, Acsbg1, Il9r, Trbc2*), and MEturquoise contained seven (*Entpd8, Krt15, Pcsk1, A430093F15Rik, Clec4g, Kdm6a, Rmrp*).

## 4 Discussion

This study uses an isogenic murine model to investigate the interactions between biological sex and maternal exposure that shape transcriptional expression and regulatory networks underlying BR under normal physiological conditions. Our lung function results confirm existing literature that shows isogenic male and female mice (differing only in sex chromosomes and hormonal milieu) exhibiting distinct BR to methacholine [47, 48, 49]. However, we find that maternal particulate exposure alone does not alter this physiological response, consistent with our previous work [25] showing that the effects of maternal exposure emerge only after postnatal exposure to an inhaled allergen. Together, these findings highlight the complex interplay between early‐life environmental exposures and later‐life lifestyle factors in shaping respiratory outcomes.

When comparing differential gene expression between isogenic male and female mice, regardless of maternal exposure, we identified 67 DEGs enriched in key biological pathways, including complement activation, hydrolase activity, fatty‐acyl‐CoA synthase activity, and cell surface localisation—suggesting sex‐specific regulation of immune function, ATP synthesis, and membrane‐associated processes under normal physiological conditions. These results align with existing murine models [50] and human clinical data in which a study of healthy Caucasian population showed that females' sera contained significantly lower levels of alternative pathway (AP) protein activity and lower levels of C3 and properdin proteins, whilst having higher factor D concentration [51]. Sex differences in complement immune system components have also been seen in disease, such as synovial fluid from patients with osteoarthritis [52], intermediate macular degeneration [53]. Sexual dimorphism in complement pathway gene expression has been reviewed across multiple disorders [54, 55], reinforcing that our findings are consistent with and extend existing literature on sex‐based differences in immune regulation. Given the well‐established role of complement signaling in airway inflammation [56] and smooth‐muscle reactivity [57], these sex‐dependent baseline differences provide a biologically plausible framework for divergent bronchial responsiveness. That is, intrinsic immune and metabolic dimorphism may prime male and female airways to respond differently to methacholine challenge, consistent with the sex‐dependent networks identified in our DEG analysis.

Moving beyond static sex differences in gene expression, we sought to determine whether gene co‐expression networks associated with BR (Rn), incorporating sex and maternal exposure as biological variables. While numerous sexually dimorphic gene networks have been identified in disease, this is the first study to demonstrate that sex modulates gene networks controlling BR under normal physiological conditions. Taken together, the biological themes emerging across MEblack, MEdarkorange, and MEturquoise point to pathways with clear relevance to bronchial responsiveness. Immune‐receptor signaling, antigen binding, and tyrosine kinase activity (MEblack) have well‐established roles shaping airway smooth‐muscle reactivity and airway inflammation during methacholine challenge [58, 59, 60]. Concurrent enrichment of mitochondrial redox and electron‐transfer pathways (MEdarkorange) highlights metabolic and oxidative processes that directly influence smooth‐muscle contractility, calcium dynamics, and airway narrowing [61, 62, 63]. Finally, the strong representation of RNA‐binding and translational‐control pathways (MEturquoise) suggests a higher‐order regulatory layer that may modulate the availability of immune and metabolic effectors in a sex‐dependent manner. Together, these network signatures provide a coherent mechanistic framework in which sex‐biased immune, metabolic, and post‐transcriptional programs converge to shape observed differences in bronchial responsiveness. Furthermore, our finding of a sex‐by‐maternal exposure interaction on BR highlights biological sex as a key modifier of the offspring's response to early‐life environmental exposures. These findings underscore the critical importance of incorporating sex as a biological variable in gene network analyses, even in the absence of overt disease, to fully understand mechanisms driving physiological variability and environmental sensitivity.

Interestingly, only a very small subset of our module eigengenes was differentially expressed. These are *Trgc4, Acsbg1, Il9r, Trbc2* from MEblack and *Entpd8, Krt15, Pcsk1, A430093F15Rik, Clec4g, Kdm6a, Rmrp* from MEturquoise. These results highlight that while module‐level eigengenes can reflect broad patterns of sex‐dependent co‐expression, not all constituent genes are differentially expressed, reinforcing that co‐expression modules capture coordinated transcriptional programs, not just differential abundance. The presence of DEGs within MEblack and MEturquoise supports their biological relevance and nominates these genes as candidate drivers of sexually dimorphic airway responses, warranting further functional investigation.

This study provides important insights into sex‐specific transcriptional regulation of BR—a known sexually dimorphic biological measure—under normal physiological conditions, using an isogenic murine model with controlled maternal exposure. However, several limitations should be considered. While most mammals follow XX/XY binary sex pathways, exceptions exist [64, 65, 66, 67], and although the mechanisms underpinning such intersex development remain an active area of investigation, they are beyond this paper's scope. While murine models offer developmental and immunological relevance, inherent species differences in lung architecture, hormone signaling, and immune responses may limit direct translatability to humans. The use of a single isogenic strain, although valuable for reducing inter‐individual variability, may underrepresent gene–environment interactions present in outbred or genetically diverse populations. Estrous staging of female mice was not performed, and culls were distributed over ~32 days, precluding cycle synchrony by co‐housing. Females were therefore likely sampled across multiple estrous phases, potentially affecting airway resistance [68] and introducing random rather than systematic variance. While hormonal fluctuations can modulate airway tone and gene expression [68], such variability would be expected to attenuate, not drive, observed sex differences. The persistence of sex‐associated physiological and transcriptomic patterns despite this uncontrolled factor suggests robustness to estrous‐related effects. Future studies incorporating estrous staging or hormonal manipulation will be valuable to disentangle ovarian influences more precisely. Moreover, this study examined transcriptional and physiological outcomes at a single postnatal time point under non‐pathological conditions. Future studies incorporating longitudinal sampling, postnatal environmental challenges (e.g., allergen exposure), and validation in human tissues will be essential to contextualize these findings within the broader landscape of sex‐specific respiratory health.

Together, these findings advance our understanding of how biological sex shapes baseline BR and the underlying transcriptional architecture of the lung. By disentangling sex‐linked gene expression patterns from maternal environmental influences under normal physiological conditions, this study provides a critical foundation for interpreting dimorphic respiratory phenotypes. These results not only reinforce the need to consider sex as a biological variable in transcriptomic and physiological studies but also highlight the value of integrative models that account for developmental exposures. As the field moves toward more personalized approaches to respiratory health, elucidating the molecular basis of sex differences will be essential for identifying tailored preventive and therapeutic strategies.

## Author Contributions

All authors approved the paper. R.Z., C.O., and B.G.O. conceived and designed the project. R.Z. performed the transcriptome and WGCNA analyses. H.C. conceived the model of maternal exposure. Y.L.C. and D.X. performed experiments. K.F.H. and H.G. collected the PM_2.5_. B.W. performed the PM_2.5_ characterization. R.Z. and C.O. prepared the paper.

## Funding

This work was supported by Wendy McCormick Research Bequest, UTS Internal. DHAC | National Health and Medical Research Council (NHMRC), 2018/GNT1158186.

## Conflicts of Interest

The authors declare no conflicts of interest.

## Supporting information


**Data S1:** Supplementary Information.

## Data Availability

Data are publicly available in a repository. The RNA‐Seq raw and processed data files generated and analyzed during the current study are available in the NCBI Gene Expression Omnibus (GEO) repository with the following accession codes.
