# Supplementary material for: Sex and the Developmental Environment Shape Molecular Networks Underlying Bronchial Responsiveness in Mice
Source: FASEB J. 2026 Feb 10;40(4):e71535. doi: 10.1096/fj.202503280R (PMC12888530; doi:10.1096/fj.202503280R)
Supplement: Supplementary file 1 — Data S1: Supplementary Information. [file FSB2-40-e71535-s001.pdf]

Data Supplement:

Supplementary Methods:

Cellular deconvolution –
We generated a lung-specific signature matrix for deconvolution using CIBERSORTx (Newman et al., 2015; Newman et al., 2019) from the adult lung subset of the Mouse Cell Differentiation Atlas (MCA 2.0; GSE176063) (Fei et al., 2022). This dataset comprised 6,940 high-quality single cells with gene-level expression mapped to the GRCm38.88 reference genome. Preprocessing was performed in R (v4.3.1). Gene identifiers were mapped to MGI symbols, deduplicated, and merged with a curated marker list, yielding 7,352 marker genes, of which 3,561 were classified as highly variable, consistent with Fei et al (2022).

The cleaned expression matrix and marker annotations were uploaded to CIBERSORTx (Create Signature Matrix module) using 32 MCA-defined lung cell types, including AT1 and AT2 epithelial cells, club and ciliated cells, endothelial and fibroblast subsets, macrophage and dendritic populations, and lymphoid lineages. CIBERSORTx was run in single-cell mode with default settings. The platform generated a gene × cell-type signature matrix by averaging expression across cells within each annotated population.

The resulting signature matrix was applied to bulk RNA-seq data in CIBERSORTx. Gene-level bulk counts were CPM-normalized per CIBERSORTx guidelines and averaged across all male (n=25) and female (n=24) samples to generate sex-specific expression profiles. Cell fractions were imputed with batch correction enabled and 100 permutations for significance testing and are reported as proportions of the whole. Validation of results was performed against previously published signature matrices (Zakarya et al., 2025).

*Newman, A., Liu, C., Green, M. et al. Robust enumeration of cell subsets from tissue expression profiles. Nat Methods 12, 453–457 (2015). https://doi.org/10.1038/nmeth.3337

*Newman, A.M., Steen, C.B., Liu, C.L. et al. Determining cell type abundance and expression from bulk tissues with digital cytometry. Nat Biotechnol 37, 773–782 (2019). https://doi.org/10.1038/s41587-019-0114-2

*Fei, L., Chen, H., Ma, L. et al. Systematic identification of cell-fate regulatory programs using a single-cell atlas of mouse development. Nat Genet 54, 1051–1061 (2022). https://doi.org/10.1038/s41588-022-01118-8

* Zakarya, R., Chan, Y.L., Wang, B. et al. Developmental air pollution exposure augments airway hyperreactivity, alters transcriptome, and DNA methylation in female adult progeny. Commun Biol 8, 400 (2025). https://doi.org/10.1038/s42003-025-07835-0

Supplementary Results:

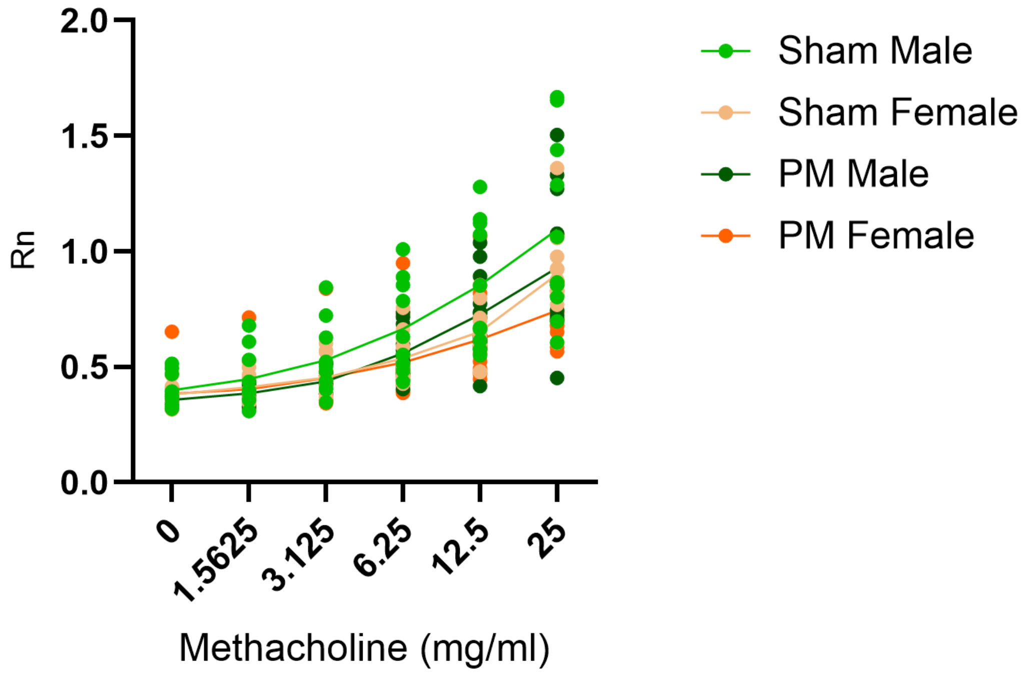

Figure S1: Spasmogen challenge and lung function testing using FlexiVent lung function measurement shows that increasing doses of methacholine increase airway resistance (Rn), with maternal exposure noted in 13-week-old female (Sham n=11; PM n=13) and male (Sham n=11; PM n=14) offspring.

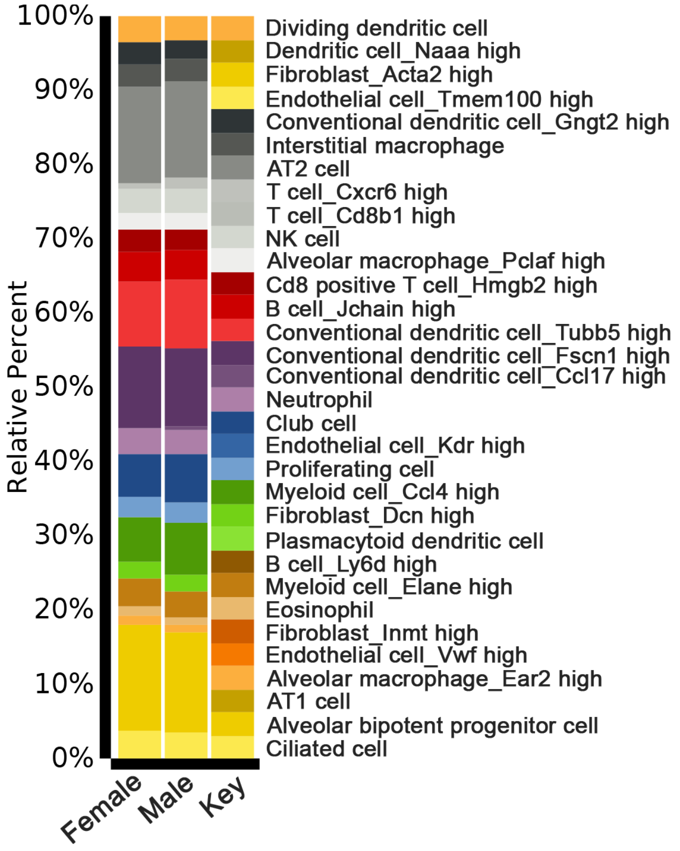

Figure S2: Cellular deconvolution of transcriptomic data. Female and Male bulk lung transcriptome (CPM) assessed with CIBERSORTX shows no significant difference in cell proportions between sexes.

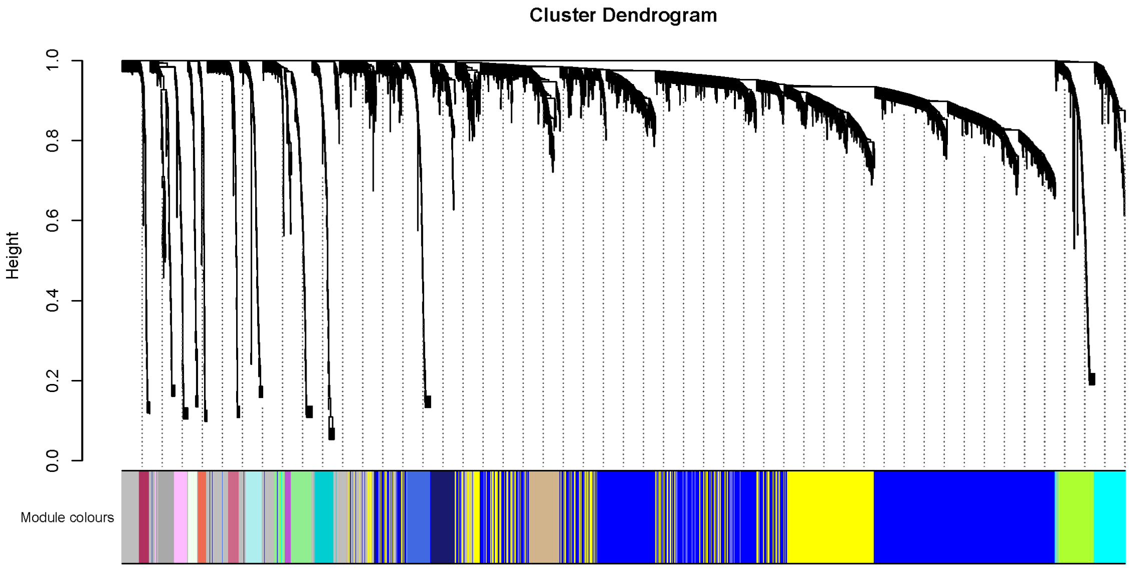

Figure S3. Hierarchical clustering dendrogram of genes based on topological overlap generated using hierarchical clustering of genes based on topological overlap matrix (TOM) dissimilarity, with average linkage. Branches of the dendrogram correspond to modules—clusters of genes with highly similar co-expression patterns—assigned distinct colours by dynamic tree cutting. These coloured modules represent the core gene co-expression networks used for downstream correlation with phenotypic traits, including sex, maternal exposure, and airway resistance (Rn)

Table S1: Univariate Analysis of Variance of methacholine challenge results data table

Between-Subjects Factors

		Value Label	N
methacholine concentration	.00000		46
	1.56250		47
	3.12500		47
	6.25000		47
	12.50000		47
	25.00000		46
Pup treatment group	1	Sham	126
	2	PM	154
Pup biological sex	5	Male	144
	6	Female	136

Tests of Between-Subjects Effects

Dependent Variable: Raw Rn response to methacholine challenge

Source	Type III Sum of Squares	df	Mean Square	F	Sig.
Corrected Model	10.748[a]	23	.467	18.991	.000
Intercept	90.279	1	90.279	3668.934	.000
Metha_conc	9.482	5	1.896	77.070	.000
Treatment_group	.213	1	.213	8.661	.004
Sex	.428	1	.428	17.374	.000
Metha_conc * Treatment_group	.122	5	.024	.994	.422
Metha_conc * Sex	.366	5	.073	2.974	.013
Treatment_group * Sex	.076	1	.076	3.106	.079
Metha_conc * Treatment_group * Sex	.010	5	.002	.084	.995
Error	6.299	256	.025		
Total	107.837	280			
Corrected Total	17.047	279			

a. R Squared = .630 (Adjusted R Squared = .597)

Table S2: Differentially expressed genes in male lungs when compared to female lungs detected by DESeq (p<0.01 and |log2 fold change| > 0.6)

Gene	Log2 Fold Change
Myh1	-6.223284245
Xist	-5.905926704
Tnnc2	-5.456155777
Alb	-3.385644436
Sostdc1	-2.553332329
Fer1l6	-2.006888151
Serpinc1	-1.594862342

Xlr4a	-1.310257792
Stab2	-1.295908689
Entpd8	-1.128260016
Krt15	-1.048729658
Ano5	-0.956404805
Pcsk1	-0.955704987
Ccr8	-0.939031601
Slitrk6	-0.83334285
A430093F15Rik	-0.80573982
Trgc4	-0.746168613
Clec4g	-0.728687882
Kdm6a	-0.667500317
A630023P12Rik	-0.659358978
Acsbg1	-0.622693717
Il9r	-0.611319721
Trbc2	-0.573515534
Osgin1	0.555022836
Gm20547	0.559725523
Fetub	0.562054276
Gpx2	0.596274853
Gpr141	0.601646066
C7	0.607622147
Serpina3c	0.609351337
Enpp6	0.688566685
Acsm1	0.710305274
Nalcn	0.717571914
Nr4a3	0.719712138
Egr3	0.724218905
Nqo1	0.787272871
Itgbl1	0.799724638
Cfb	0.815614045
Lars2	0.83789432
Trim67	0.849140167
Egr1	0.85941422
Pla1a	0.921924293
Gm20721	0.945828438
Slc7a11	0.990168393
Acsm3	1.016905427
Rmrp	1.031566858
Rmrp	1.031566858
Ces1f	1.034624219
Cd177	1.158106923

Serpina3m	1.248533607
Tnfsf11	1.307978511
Rpph1	1.334112883
Ces1g	1.334346533
Arc	1.443974972
Gm19950	2.106935024
Them5	2.353266954
Gm48314	2.738600731
Gm43417	2.80338788
Gm49838	3.031541824
Kcnj16	3.492312193
Gm42907	3.570821047
Gm29650	8.056103706
Uty	10.035532
Ddx3y	10.93411827
Kdm5d	10.95735073
Eif2s3y	11.46543312
Kap	24.78086472
